# Necrotizing fasciitis after placement of intraperitoneal catheter^☆^

**DOI:** 10.1016/j.gynor.2013.04.005

**Published:** 2013-05-04

**Authors:** Jori S. Carter, Sarah L. Hutto, Javariah I. Asghar, Deanna G.K. Teoh

**Affiliations:** a University of Minnesota, Division of Gynecologic Oncology, USA; b University of Minnesota, Department of Surgery, USA

**Keywords:** Intraperitoneal catheter, Necrotizing fasciitis, Intraperitoneal catheter complications

## Abstract

• Necrotizing fasciitis has not previously been reported in association with placement of intraperitoneal port at time of cytoreductive surgery.
• Consensus is lacking regarding the placement of intraperitoneal ports at the time of bowel surgery.
• Delayed placement of intraperitoneal ports may be considered in patients undergoing bowel resection.

## Introduction

Intraperitoneal (IP) chemotherapy has a proven survival advantage in patients with optimally cytoreduced advanced epithelial ovarian cancer (Armstrong et al., 2006). Placement of IP catheters can be performed at the time of optimal cytoreductive surgery (Davidson et al., 1991; Walker et al., 2006). Reported here is the case of a woman with stage IIIC serous adenocarcinoma of primary peritoneal origin who underwent optimal cytoreductive surgery and IP catheter placement, who was readmitted on post-operative day 10 with necrotizing fasciitis of her anterior abdominal wall originating at the IP catheter site.

## Case

A 63-year-old nulligravid female with a past medical history significant for moderately controlled type II diabetes (glycosylated hemoglobin 7.5%), hypertension, hyperlipidemia, and morbid obesity (BMI 44 kg/m^2^) presented to her primary care physician with complaints of two weeks of lower abdominal pain, bloating, early satiety, and nausea. She was treated with antibiotics for presumed diverticulitis for 2 weeks without resolution of symptoms. A CT scan of the abdomen and pelvis revealed inflammatory changes in the pelvis surrounding the bilateral adnexa and sigmoid colon with multiple colonic diverticula present suggestive of uncomplicated diverticulitis, omental nodularity, and soft tissue prominence in the left adnexa. Pelvic ultrasound showed a 5 cm calcified fibroid with submucosal extension, and the ovaries could not be visualized. A serum CA125 level was elevated to 907 U/mL. She was referred to the Gynecologic Oncology clinic for further evaluation and scheduled for surgery. The patient underwent a diagnostic laparoscopy, which revealed omental caking, moderate straw-colored ascites, and bowel adhesions to the anterior abdominal wall. Due to findings concerning for malignancy, the procedure was converted to laparotomy and optimal cytoreduction to < 1 cm residual disease was performed, including total abdominal hysterectomy, bilateral salpingo-oophorectomy, infragastric omentectomy, appendectomy, resection of proximal descending colon with side-to-side re-anastomosis. IP port was placed at the time of surgery. Surgical pathology showed a stage IIIC high-grade serous adenocarcinoma of primary peritoneal origin.

The immediate post-operative course was uncomplicated and she was discharged home on post-operative day 5. On post-operative day 10, the patient presented to clinic with complaints of increased left sided abdominal pain that she described as a constant, pulling sensation centralized around the IP port site. She also reported brown, watery discharge from this site. She denied fevers or chills, or other signs of infection. Evaluation was significant for significant pain at the port site without surrounding erythema, and hypotension with blood pressure 79/38 mm Hg, normal pulse of 95 bpm. The patient was taken to the Emergency Department for further evaluation.

**Fig. 1:**
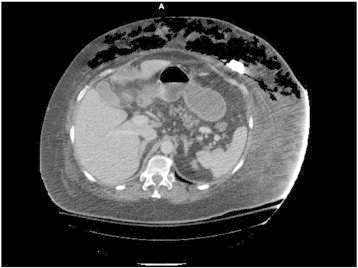
Computed tomography image of extensive subcutaneous emphysema surrounding the IP catheter.

In the Emergency Department, the patient was started on norephinephrine for blood pressure support and broad-spectrum antibiotics with piperacillin-tazobactam and vancomycin. CT scan of the abdomen and pelvis showed massive subcutaneous emphysema present within the abdominal wall tracking from the IP port site concerning for necrotizing fasciitis, without evidence of anastomotic leak or intra-abdominal abscess (Fig. 1). General surgery and intensive care teams were consulted and given the concern for necrotizing fasciitis and severe sepsis, she was taken to the operating room for debridement.

**Fig. 2:**
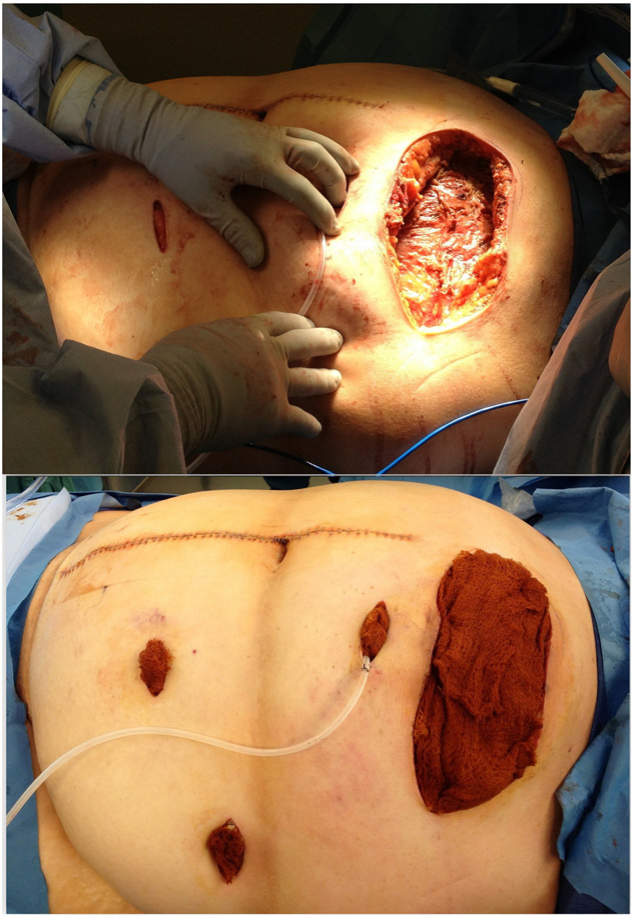
Surgical debridement of subcutaneous tissue and anterior fascia surrounding the IP catheter site was performed.

Three subcutaneous incisions were made in the left abdominal wall initially, which did not show evidence of infection or necrotic tissue. Removal of the IP port then revealed necrotizing fasciitis at the left upper quadrant IP port reservoir site at the level of the deep subcutaneous tissue and fascia; superficial tissue showed no evidence of necrosis. Radical debridement of the abdominal wall, including anterior fascia, muscle, skin, and soft tissue was performed over a 20 cm by 10 cm area. A subcutaneous drain was placed, and the wounds were packed open with betadyne-soaked Kerlix gauze (Fig. 2). On hospital day 4, a wound V.A.C.® Therapy Dressing (KCI, San Antonio TX) was placed on the 20 cm by 10 cm wound, and the three small wounds were packed with gauze. Wound cultures grew *Enterobacter cloacae*, *Streptococcus viridans*, and greater than four anaerobic organisms, which were not speciated by the laboratory. Two sets of blood cultures were positive for growth of *Bacteroides fragilis*. Broad spectrum antibiotics were continued for 7 days, and then narrowed to levofloxacin for the subsequent 7 days. The patient was weaned off of pressors after hospital day 7, and discharged to a transitional care facility on hospital day 11. The wounds all completely healed within 8 weeks.

One week after discharge (28 days after her initial cytoreductive surgery) she presented to clinic with fecal material draining from the vagina. CT scan showed an anastomotic leak with fistula tract to the vagina. Review of the CT performed on postoperative day 10 revealed a small pocket of extraluminal air in the presumed ascites fluid, raising suspicion of a small anastomotic leak, which had not been identified on the initial CT read. Due to the patient's poor nutrition (albumin 1.7 g/dL), surgical management was deferred and a percutaneous drain was placed. After 15 weeks, the fistula had completely resolved, and the drain was removed. She has completed 6 cycles of primary chemotherapy with IV carboplatin and paclitaxel without difficulty. CA-125 prior to starting chemotherapy (12 weeks after cytoreductive surgery) had decreased to 123 U/mL, and abdomen and pelvic CT performed to evaluate the anastamotic leak one week after initiating chemotherapy showed no radiographic evidence of disease. CA-125 normalized after the first cycle of chemotherapy, and was 7 U/mL at the completion of chemotherapy.

## Discussion

Necrotizing fasciitis is a progressive, polymicrobial, postoperative infection of the muscular fascia with associated necrosis that was first described by Brewer and Meleney in 1926 (Brewer and Meleny, 1926). Non-necrotizing infections involve the skin (epidermis and dermis) as well as the subcutaneous tissue and usually respond to antimicrobial treatment alone. Necrotizing soft tissue infections, however, involve the skin, subcutaneous tissue as well as muscle and fascia, and require urgent operative debridement. While it is critical to distinguish between these two types of soft tissue infections, it is often difficult to discern this difference in the absence of obvious signs of an underlying necrotizing soft tissue infection.

Patients often present with pain, possibly associated with fever or cellulitis or crepitus. A thorough history and physical examination are critical. Risk factors include diabetes, peripheral vascular disease, immune compromise, and recent surgery. Approximately 5% of cases occur in the setting of a postoperative infection, in both contaminated and clean–contaminated operations (Wong et al., 2003). On examination, pain is often found to be out of proportion to the presenting symptoms with tenderness beyond the margins of any presenting erythema or subcutaneous crepitus. Helpful imaging studies in the absence of overt signs of an infection include a CT scan or an MRI. The key components of the initial treatment for necrotizing fasciitis include adequate fluid resuscitation with electrolyte correction, physiologic support of any failing organs, broad-spectrum antimicrobial treatment, and supportive care. The cornerstone of this treatment remains urgent and thorough debridement of any necrotic tissue.

Multiple studies have shown that administration of IP chemotherapy significantly improves overall survival, and placement of IP catheters for IP chemotherapy following optimal cytoreduction of stage III ovarian cancer has become a standard procedure for many gynecologic oncologists. The most common catheter complications are port malfunction (5–9%) and infection (4–10%) (Davidson et al., 1991; Walker et al., 2006; Landrum et al., 2008; Lesnok et al., 2010; Makhija et al., 2001). This is the first report of necrotizing fasciitis following placement of an IP catheter at the time of cytoreductive surgery (after PubMed search of any combination of necrotizing fasciitis, intraperitoneal catheter or port, ovarian cancer, or cytoreductive surgery). Necrotizing fasciitis is reported in one case of delayed IP catheter placed under fluoroscopy by interventional radiology, which was managed by surgical debridement (Rundback et al., 1994).

IP catheter placement at the time of bowel resection is controversial. Authors have suggested avoiding placement of IP catheters at the time of large bowel surgery (Makhija et al., 2001), however the data has not proven that this would reduce the rate of catheter complications. Analysis of GOG 172 data suggests that delayed placement of IP catheters did not decrease the likelihood of complications (Walker et al., 2006). A retrospective study performed at our institution of optimally cytoreduced patients (82% rate of optimal cytoreduction to < 1 cm, of which 22% involved bowel surgery other than appendectomy) did not reveal a significantly increased complication rate when IP catheters were placed at the time of bowel surgery (14.3% versus 25% in patients with IP ports placed at the time of bowel surgery versus patients who did not have IP ports placed, respectively, RR 0.81, 95% CI 0.21, 2.58, n = 14). Rates of bowel surgery did not differ among those who received and did not receive an IP port at the time of optimal cytoreductive surgery (p = 0.40). Complications of IP ports placed at the time of bowel surgery included inflow obstruction (n = 1) and extravasation (n = 1) (Carter et al., 2011).

This case contributes to the body of literature describing complications of IP catheters. It remains unclear if necrotizing fasciitis in this case was associated with the large bowel resection or coincidence. Delayed placement of IP catheters may be considered if large bowel surgery is performed at the time of cytoreduction, although data is not definitive that complication rate will be reduced with delayed placement. Careful consideration of the risks and benefits of primary versus delayed placement IP catheters should be performed in cases of cytoreductive surgery, especially at the time of large bowel resection with re-anastomosis.

## Conflict of interest statement

The authors declare that there are no conflicts of interest.
